# LEF1/Id3/HRAS axis promotes the tumorigenesis and progression of esophageal squamous cell carcinoma

**DOI:** 10.7150/ijbs.47035

**Published:** 2020-06-29

**Authors:** Xinyu Wang, Yue Zhao, Xiang Fei, Qijue Lu, Yang Li, Yang Yuan, Chaojing Lu, Chunguang Li, Hezhong Chen

**Affiliations:** 1 Department of Thoracic Surgery, Changhai Hospital, Second Military Medical University, Shanghai 200433, China;; 2 Department of Cardiovascular Surgery, Changhai Hospital, Second Military Medical University, Shanghai 200433, China.

**Keywords:** LEF1, Id3, HRAS, esophageal squamous cell carcinoma, MAPK signaling pathway.

## Abstract

Our previous study demonstrated that lymphoid enhancer-binding factor 1 (LEF1) could promote the progression of esophageal squamous cell carcinoma (ESCC). However, the regulatory mechanism of LEF1 was not clear thoroughly. Herein, we continued to explore the downstream mechanism of LEF1 in ESCC. In this study, we applied western blotting, quantitative real-time polymerase chain reaction (qRT-PCR), immunohistochemistry, RNA-Seq analysis, a luciferase reporter assay, chromatin immunoprecipitation (ChIP), bioinformatics analysis, and a series of functional assays *in vitro* and *in vivo*. The results demonstrated that LEF1 regulated directly the expression of Id3. Id3 was highly expressed in ESCC tissues and correlated with histologic differentiation (p=0.011), pT stage (p<0.01) and AJCC stage (p<0.01) in ESCC patients. Moreover, Id3 could serve as a prognostic factor of ESCC. By various functional experiments, overexpression of Id3 promoted the proliferation, migration, invasion, EMT, and tumorgenicity. Mechanistically, Id3 could regulate ERK/MAPK signaling pathway via activating HRAS to perform its biological function. Furthermore, activating ERK/MAPK signaling pathway promoted the expression of Id3 gene in turn, indicating that a positive regulatory loop between Id3 and ERK/MAPK pathway may exist in ESCC. In summary, LEF1/Id3/HRAS axis could promote the tumorigenesis and progression of ESCC via activating ERK/MAPK signaling pathway. Targeting this cascade may provide a valid antitumor strategy to delay ESCC progress.

## Introduction

Esophageal cancer (EC) is the 8th most common cancer and the 6th leading cause of death among all cancers worldwide 1. Esophageal squamous cell carcinoma (ESCC) is the predominate EC subtype in Asian countries, especially in China. Although much progress has been made in treating ESCC, the survival of patients is still beyond satisfaction 2. A combination of surgery with chemoradiotherapy acquired limited improvement of 5-year survival rate, suggesting that traditional therapy has reached a plateau. Thus, it is urgent to identify effective therapeutic targets to delay ESCC progress.

As a key regulator of the Wnt/β-catenin signaling pathway, lymphoid enhancer-binding factor 1 (LEF1) drives the expression of genes involved in cell proliferation, embryonic and organ development in normal cells 3. Increasing studies have unveiled that dysregulation of LEF1 is implicated in the progression of multiple cancers, such as breast cancer, colon cancer, prostate cancer, et al 4-6. Our previous studies showed that LEF1 was highly expressed in ESCC tissues and it could promote proliferation, migration, invasion and epithelial-mesenchymal transition (EMT) of ESCC cells 7,8. More recently, we found LEF1 could promote the stem-like phenotype and the tumorigenicity in ESCC 9. However, the downstream regulatory mechanism of LEF1 responsible for its oncogenic role remains unclear and is worth further exploration.

The inhibitor of DNA binding (Id) protein includes Id1, Id2, Id3, and Id4 members, function as inhibitors of basic helix-loop-helix (bHLH) transcription factors 10. As vital regulators of cell cycle, cell growth and differentiation, Id proteins are increasingly observed involving in various tumor-related processes, such as chemotherapeutic drug resistance 11, stem cell features 12, metabolic reprogramming 13, EMT 14, angiogenesis and metastasis 15. However, studies about the role of Id proteins in ESCC were limited, especially for Id3.

The RAS family of proteins (KRAS, HRAS, and NRAS) are well-known oncogenic drivers in various cancers 16. Activated RAS triggers cellular signaling cascades and biological processes through activating RAS-MEK-ERK (MAPK) pathway 17. Aberrant activation of ERK/MAPK pathway was observed in various cancers. Therefore, targeting the ERK/MAPK signaling pathway is a promising therapeutic target 18,19.

In this study, we continued to explore the downstream mechanism of LEF1 in ESCC. We demonstrated that LEF1 regulated directly the expression of Id3. Id3 was highly expressed in ESCC tissues and correlated with poor prognosis of ESCC patients. Overexpression of Id3 promoted the proliferation, migration, invasion, EMT, and tumorgenicity. Mechanistically, Id3 could regulate ERK/MAPK signaling pathway via activating HRAS to perform its biological function. Furthermore, activating ERK/MAPK pathway promoted the expression of Id3 gene in turn, indicating that a positive regulatory loop between Id3 and ERK/MAPK pathway may exist in ESCC. These findings suggest that LEF1/Id3/HRAS axis plays an important role in ESCC progression and predicts promising targets for treating ESCC.

## Material and Methods

### Clinical tissue and specimens

Fourteen fresh ESCC tissues were obtained in operation from Changhai Hospital, affiliated to Second Military Medical University (Shanghai, China). 92 paraffin-embedded samples used in this study were obtained from patients who were diagnosed with primary ESCC and received esophagectomy from 2012 to 2013 at Changhai Hospital. None of these patients had a history of preoperative chemotherapy or radiotherapy. Overall survival (OS) was defined as the time interval from surgery to death or last observation. Ethics approval for the study was given by the Ethics Committee of Changhai Hospital and written informed consent was obtained from all patients.

### Cell lines and Reagents

The human ESCC cells (Eca109 and TE1) were purchased from the Shanghai Cell Bank (Shanghai, China). All cell lines cultured in DMEM (Gibco, CA, USA) supplemented with 10% FBS (Gibco-BRL) and 1% antibiotics (penicillin and streptomycin; HyClone Laboratories, Inc., USA).

TPA (CST, 4174) and U0126 (CST, 9903) were used to active or inactive ERK/MAPK signaling pathway. DMSO (CST, 12611) was used for a negative control.

### Immunohistochemistry (IHC)

Two independent pathologists performed IHC using a modified Histo-score (H-score). The 92 ESCC tissue slides were incubated with Id3 (1:100, Santa Cruz, sc-56712) and LEF1(1:200, Abcam, ab137876) antibodies. The detailed immunostaining staining procedures were performed with reference to our previous study 8. Briefly, the proportion of positively stained cells was scored as 0-100% and the intensity score were scored as 0 (negative), 1+(weakly positive), 2+(moderately positive), or 3+ (strongly positive). A final score was then calculated by multiplying these two scores.

### RNA extraction and quantitative RT-PCR (qRT-PCR)

qRT-PCR was employed to measure the mRNA expression level. Briefly, TRIzol (Invitrogen) was used for total RNA extraction and PrimeScript RT Reagent Kit (TaKaRa) was used for synthesizing the cDNA. Afterwards, SYBR Premix EX Taq (TaKaRa) was used for quantitative real-time polymerase chain reaction. Relative changes of gene expression were determined by 2^-△△Ct^. Primer sequences are listed as followed and all experiments were performed in triplicate.

HRAS:

5'-AGCAGGTGGTCATTGATGG-3'(F), 5'-GTTTGATCTGCTCCCTGTACT-3′(R);

DUSP16:

5'- GCCCATGAGATGATTGGAACTC-3'(F),

5'- CGGCTATCAATTAGCAGCACTTT-3′(R);

C-JUN:

5'- GGAGTGTCCAGAGAGCCTTG-3'(F),

5'- GAAAGGCTTGCAAAAGTTCG-3′(R);

C-FOS:

5'- TTACTACCACTCACCCGCAG-3'(F),

5'- AGTGACCGTGGGAATGAAGT-3′(R);

GAPDH:

5'-GGAGCGAGATCCCTCCAAAAT-3'(F) 5'-GGCTGTTGTCATACTTCTCATGG-3′(R).

### Western blotting

Whole six-well cells were lysed in 250ul 0.1% SDS and 10ul 1mM PMSF. Protein extracts were separated by SDS-PAGE and analyzed using the following primary antibodies: Id1 (Santa Cruz, sc-374287), Id3 (Santa Cruz, sc-56712), HRAS (Santa Cruz, sc-29), Vimentin (Santa Cruz, sc-6260), p-MEK (CST, 9154), p-ERK (CST, 4370), MEK (CST, 4694), ERK (CST, 4695), Snail (CST, 3879), Slug (CST, 9585), Slug (CST, 9585), MMP2 (CST, 4022), MMP9 (CST, 3852) , C-FOS (Proteintech, 66590-1-Ig), C-JUN (Proteintech, 24909-1-AP), LEF1(Abcam, ab137872), DUSP16 (Abcam, ab181088), E-cadherin(Abcam, ab40772), N-cadherin(Abcam, ab18203) and GADPH antibody (Abcam, ab8245). The next day, the membranes were incubated with secondary antibodies (CST,7076,7074) at room temperature for 2 hours. All results were repeated 3 times independently.

### Bioinformatics analysis

We utilized the Oncomine (https://www.oncomine.org/resource/login.html) and UALCAN (http://ualcan.path.uab.edu/analysis.html) database for detecting the expression level of genes. JASPAR (http://jaspar.genereg.net/) and PROMO (http://alggen.lsi.upc.es/) database were used to analysis and predicted the binding sites of gene promoters.

### Cell proliferation assays and colony formation assay

Cell proliferation was measured by Cell Counting Kit-8 (CCK-8, bimake, USA). Briefly, infected ESCC cells were seeded in 96-well plates (5000 cells/well) and cultured for 4 days. The absorbance was measured at 450 nm. For the colony formation assay, ESCC cells were seeded in 6-well plates. After incubation for 10 days (Eca109) or 14 days (TE1), cells were fixed with 4% paraformaldehyde and stained with 1% crystal violet. The number of colonies containing ≥50 cells was counted.

### Wound healing assay

Wound healing assay was used to assess the cell mobility. Briefly, ESCC cells were seeded in six-well plates, counting the cell density for about 90% after 24h. Multiple scratches were generated with 200ul pipette tips. Subsequently, old culture solution was moved out and the plates were washed with PBS. Instead, the cells were filled with 2ml serum-free medium. Typical images were photographed at 0h and 48h under an inverted microscope (Olympus).

### Cell migration and invasion assay

To assess the migration and invasion ability, 24-well transwell chambers (Corning) with Matrigel (Corning, Bedford, MA, USA) coating or not were used in the experiment. Briefly, approximately 1*10^5^ cells were resuspended in 300ul serum-free DMEM and seeded into the upper chambers, whereas the bottom chamber was filled with 500uL 20% FBS medium. After incubating 24 hours, the migrated/invaded cells in lower chamber were fixed with 95% paraformaldehyde for 30 minutes and stained with 1% crystal violet for 30 minutes. The cells were counted by Image J software (Rawak Software Inc., Stuttgart, Germany).

### Construction of overexpression and knockdown of genes

Lentiviral vectors expressing Id3 gene (ovId3) and shRNA targeting Id3 (shId3) were designed by HeYuan Bio-technology Company, Shanghai, China. HRAS knockdown plasmids and negative control plasmids were transfected by Lipofectamine 2000 (Invitrogen; Thermo Fisher Scientific Inc., USA). Cells were harvested at 2 days after transfection for RNA analysis and 3 days after transfection for protein analysis.

### Xenograft Mice

Male BALB/c nude mice (six weeks old) were provided by Shanghai Experimental Center (Shanghai, China). Approximately 4×10^6^ ovId3 (shId3) Eca109 cells and control cells resuspended in 150ul PBS were subcutaneously injected into the right dorsal flank of per mouse. Nude mice were observed for 25-35 days and sacrificed. Tumor volume was measured every 5 days and evaluated with the standard formula: V (cm^ 3^) = 1/2×L (length) × W(width)^2^.

### RNA-seq

OvId3 Eca109 cells and negative control cells (three samples per group) were collected for RNA-seq analysis by Shanghai Novelbio corporation (Shanghai, China). Detailed procedures were carried out according to our previous study 8.

### Luciferase reporter assay

Cells were seeded in 24-well plates, followed by transient co-transfection with vector or LEF1 plasmids, and Id3 promoters using Lipofectamine™ 2000 (Invitrogen). After 48h, luciferase activities were measured (Promega).

### Chromatin immunoprecipitation (ChIP) assays

SimpleChIP® Enzymatic Chromatin IP Kit (Magnetic Beads, CST, 9003) was applied for ChIP assay. All procedures were conducted by the manufacturer's instructions. Briefly, ESCC cells (4×10^6^) were cross-linked by 1% formaldehyde. Chromatin was digested with 0.5 µl micrococcal nuclease per 4×10^6^ cells. 10ul LEF1 antibody (Abcam, ab137872), 1ul normal rabbit IgG (CST, 2729), and 10ul Histone H3 Rabbit mAb (CST, 4620) were incubated with samples at 4 °C for overnight. Then we eluted the chromatin from antibody/protein G magnetic beads and reversed the cross-links. Immunoprecipitated DNA was amplified by PCR using specific primers. The primer sequences for Id3 were 5′-TGTCCTGACACCTCCAGAACG-3′ (forward), and 5′-TCACAGTCCTTCGCTCCTGA-3′ (reverse).

### Statistical analysis

IBM SSPS 24 (IBM Corp., Armonk, NY, USA) was employed to analyze the data. Student's t-test was used to determine the differences between groups and two-tailed ANOVA test was applied in case of multiple groups. Correlations of two genes were analyzed by Pearson's correlation. Kaplan-Meier curves were used to compare the OS between groups. Multivariate analysis was employed to determine independent factors affecting the prognosis of patients. The level of statistical significance was set at p < 0.05(*) or p < 0.01(**).

## Results

### LEF1 directly bound to the human Id3 gene promoter

**Figure 1:**
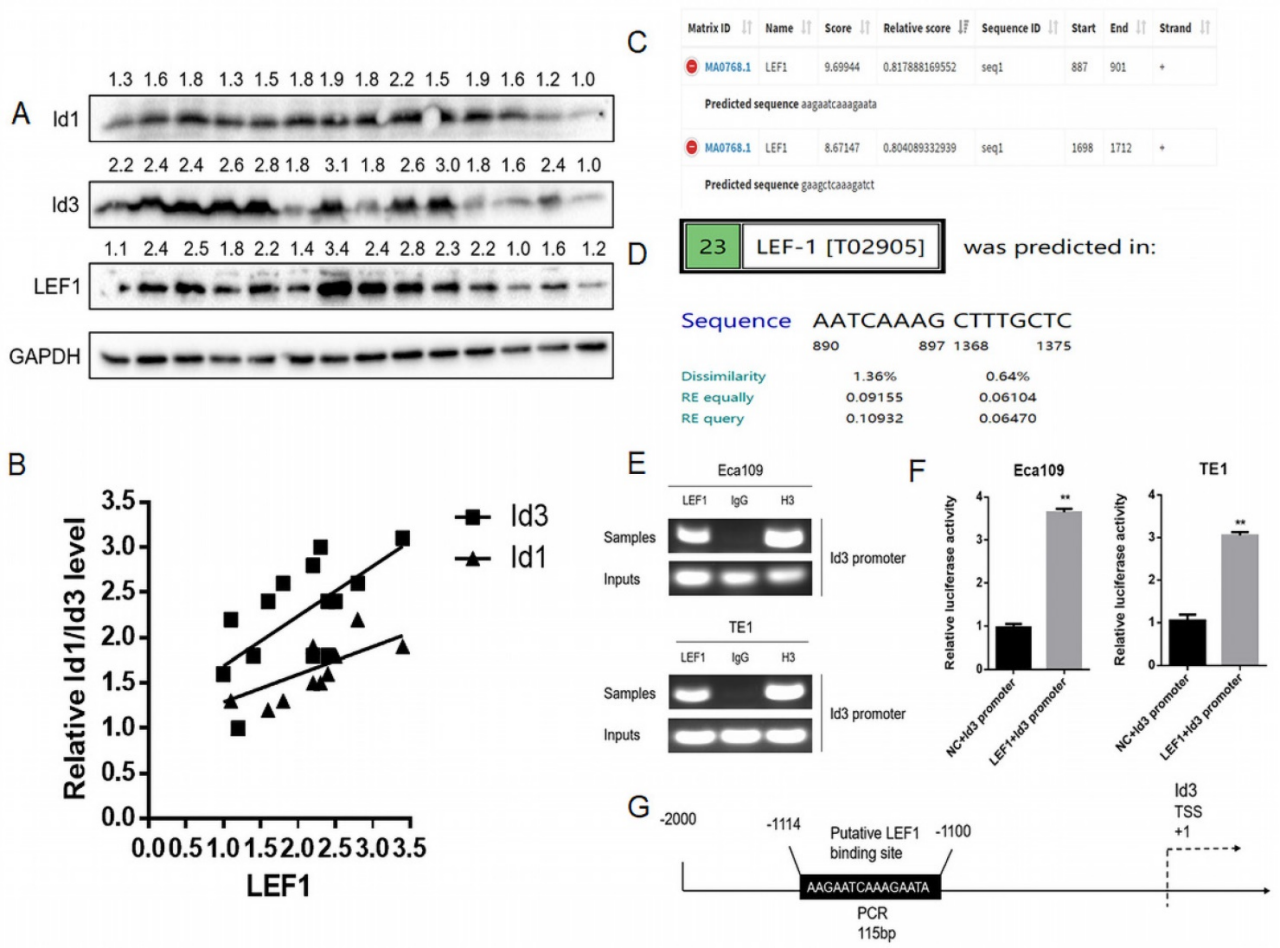
LEF1 directly bound to the human Id3 gene promoter. (A, B) LEF1 was positively correlated with the expression both of Id1 and Id3 in the term of protein level. (C, D) Potential LEF1 binding sites to the Id3 were identified by two public databases. (E) Assessment of LEF1 binding to the Id3 sequence in Eca109 and TE1 cells was performed by ChIP. (F) The luciferase activities of the constructs of the Id3 sequence in Eca109 and TE1 cells transfected with either LEF1 or controls. (G) Schematic diagram of potential LEF1 binding site in the Id3 sequence. *P<0.05, **P<0.01

Based on our previous RNA-Seq data and qRT-PCR results, both Id1 and Id3 were increased significantly after LEF1 was overexpressed 8. Although Id1 expression showed the highest fold change on transcriptional level, protein changes of the two Id genes have not measured. Thus, we examined the association between LEF1 and Id1/Id3 expression in 14 freshly ESCC specimens. According to the WB results, we discovered that LEF1 was positively correlated with both of Id1 and Id3 expression, especially a higher correlation of LEF1-Id3 was observed (Fig. 1A, 1B). Meanwhile, JASPAR and PROMO database also predicted that the Id3 gene promoters contained several potential binding sites of LEF1 (Fig. 1C, 1D). All of above indicated that LEF1 may bind to the promoter area of Id3. Next, we used a luciferase reporter assay and ChIP-qPCR assay to verify it and the experimental results were consistent with our presumption (Fig. 1E-G). Therefore, Id3 was identified as a target gene of LEF1 and selected for further study.

### Correlation between LEF1 and Id3 expression in ESCC tissues, and their associations with clinicopathological characteristics

**Figure 2:**
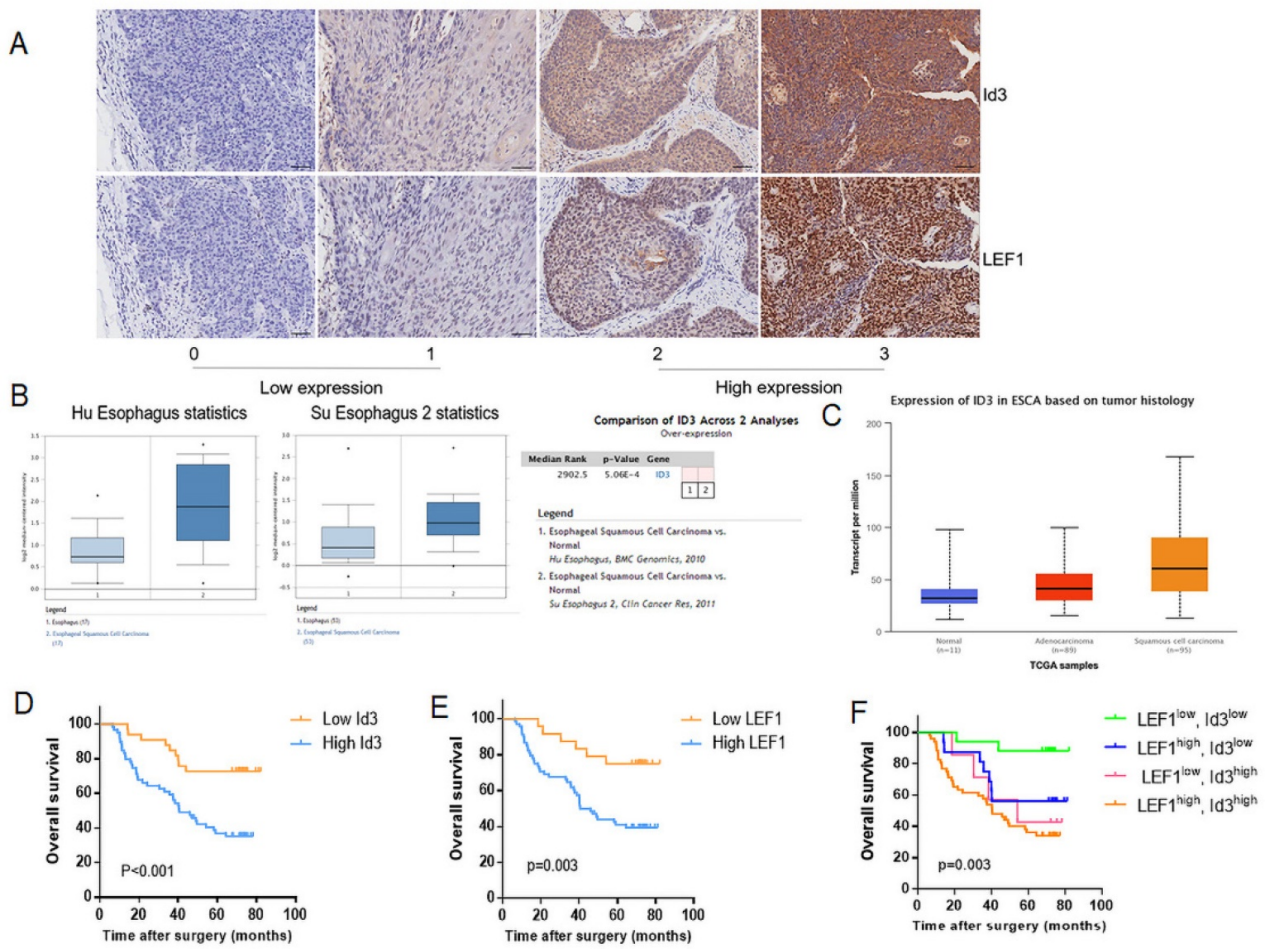
Correlation between LEF1 and Id3 expression in ESCC tissues, and their associations with clinicopathological characteristics. (A) Representative staining intensity of Id3 by immunohistochemistry analysis (×200, scale bar=100μm). (B, C) Oncomine (B) and UALCAN (C) data showed that Id3 was highly expressed in ESCC tissues. (D, E) Overall survival curve of 92 patients with high expression and low expression of Id3 (D, P<0.001) or LEF1 (E, P=0.003) expression level. (F) Comparison of the overall survival among four groups (P=0.003).

Firstly, we determined the relationship between LEF1 and Id3 expression in 92 patient specimens. Consecutive section of IHC showed a potential association between LEF1 and Id3 expression (Fig. 2A). Besides, the percentage of patients with high Id3 expression was 76.5% (52/68) in the group of high LEF1 expression, indicating a tight association between LEF1 and Id3 expression (Table 1). Next, we detected the expression of Id3 in two public databases, Oncomine and UALCAN. As shown in Fig. 2B, 2C, Id3 expression in ESCC tissues was higher than that in normal tissues. Then we investigated whether Id3 was related to cancer characteristics using the UALCAN database. As shown in Fig S1A, patients with poorly differentiated esophageal cancer tended to have the highest Id3 expression level. Moreover, the data shown in Fig S1B indicated that patients with the lymph node metastasis tended to have higher Id3 expression than those without.

Subsequently, we investigated the association between Id3 and clinicopathological features of ESCC patients. As shown in Table 2, the obvious higher expression level of Id3 protein was detected in ESCC tumor tissues compared with adjacent normal tissues (p<0.01). Table 3 showed the correlation between Id3 expression and clinicopathological features of ESCC patients. We found that the Id3 expression was associated closely with histologic differentiation (p=0.011), pT stage (p<0.01) and AJCC stage (p<0.01). However, no significant difference was observed between Id3 expression and age (p=0.500), sex (p=0.572), tumor location (p=0.907) or pN stage (p=0.074).

Based on the association between LEF1 and Id3 expression, all patients were divided into 4 groups: LEF1^high^ and Id3^high^ group (n = 52); LEF1^high^ and Id3^low^ group (n = 16); LEF1^low^ and Id3^high^ group (n = 7); LEF1^low^ and Id3^low^ group (n = 17). Kaplan-Meier analysis demonstrated that patients with high Id3 expression had poorer overall survival (OS) than those with low Id3 expression (p=0.007; Fig. 2D). Meanwhile, patients with high LEF1 expression group also showed more unfavorable prognosis than their counterparts (Fig. 2E). Patients with LEF1^high^ and Id3^high^ expression were associated with the poorest survival time (Fig. 2F). By univariate Cox analysis, differentiation, pN stage and Id3 expression were risk factors affecting OS (p=0.035, p=0.025; p=0.002, respectively). Multivariate Cox analysis revealed that Id3 expression was the only independent factor affecting the OS (p=0.007, Table 4).

### Id3 regulated proliferation, migration, and invasion in human ESCC cells

**Figure 3:**
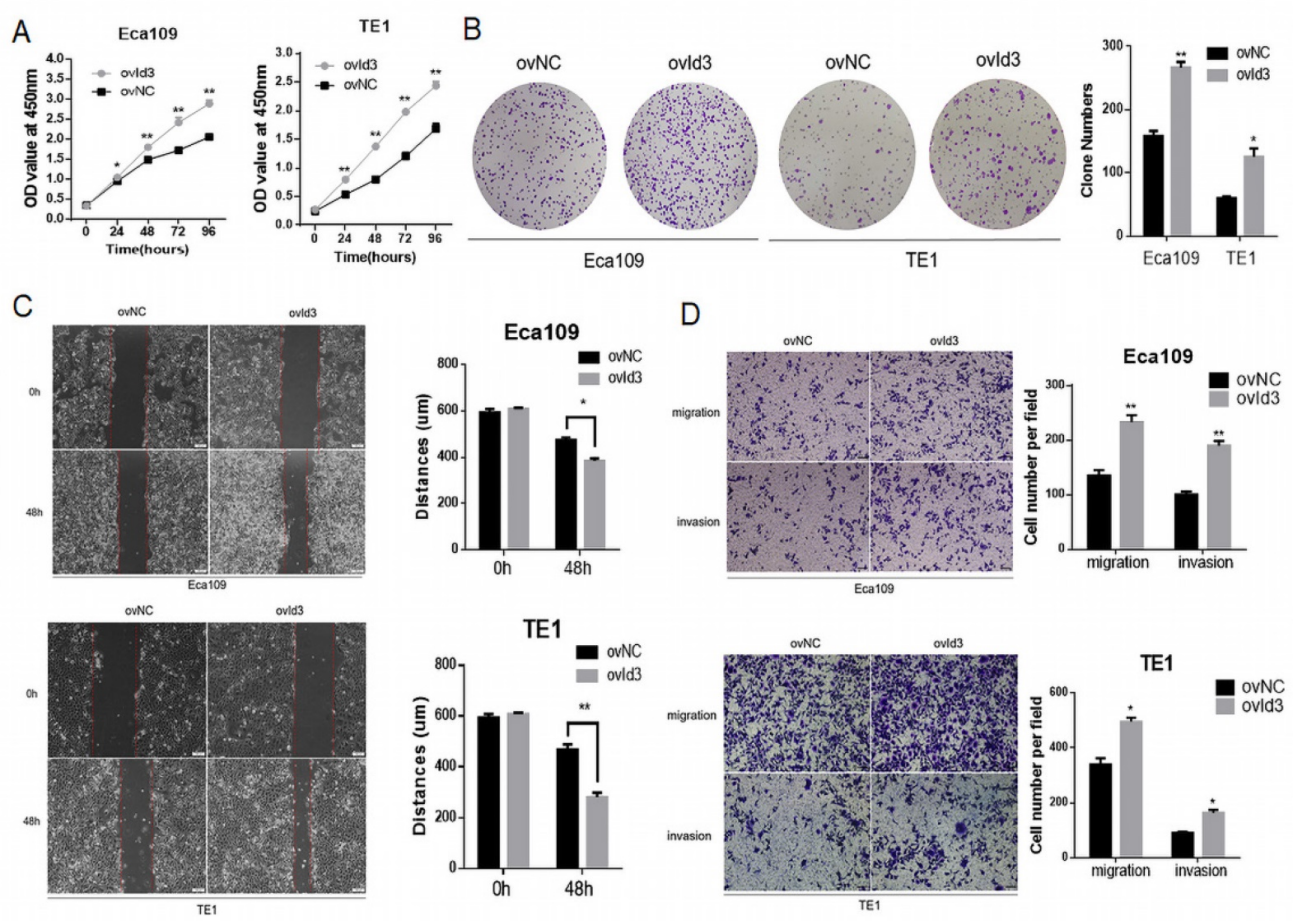
Id3 promoted proliferation, migration, and invasion in Human ESCC cells. (A) Both Eca109 and TE1 cells viability was increased after transfected with overexpressed Id3, detected by CCK-8 assay. (B) Proliferative ability of Eca109 and TE1 cells was significantly increased in ovId3 cells by clone formation assay. (C) Overexpression of Id3 promoted ESCC cell migration *in vitro* as assessed by the wound healing assay (scale bar, 100μm). (D) Overexpression of Id3 promoted ESCC cell migratory and invasive abilities *in vitro* as assessed by the transwell assay (scale bar, 100 μm) *P<0.05, **P<0.01.

To evaluate the functional effects of Id3 on ESCC cells, we constructed ESCC cells of stably express Id3 (ovId3). Empty vector-transfected cells were used as controls (ovNC). The overexpression efficiency of Id3 in ESCC cells was satisfactory detected by qRT-PCR and WB analysis (Fig. S2A). The CCK-8 assay and colony formation assay demonstrated that Id3 overexpression promoted the proliferation of ESCC cells (Fig. 3A, 3B). To explore the impacts of Id3 on cell motility, we performed the wound healing assay. As shown in Fig. 3C, the distance of gap in Id3 overexpression group was shorter than in their corresponding control group. Subsequently, we performed Transwell migration and Matrigel invasion assays. Results showed that the migratory and invasive abilities of ESCC cells were enhanced obviously in Id3 overexpression group compared to control group (Fig. 3D).

**Figure 4:**
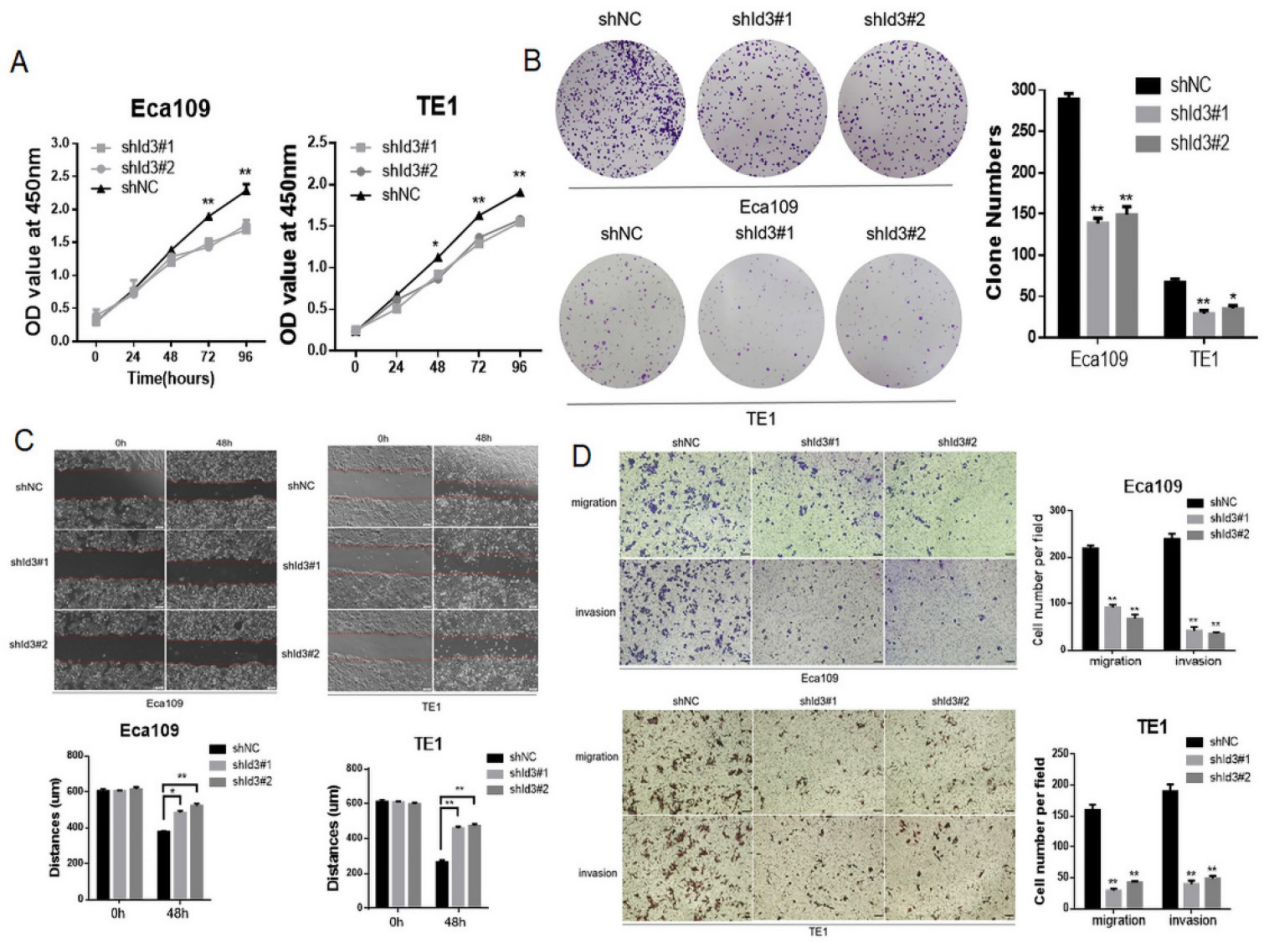
Silencing Id3 inhibited proliferation, migration, and invasion in Human ESCC cells. (A) Both Eca109 and TE1 cells viability was suppressed after transfected with shRNA-Id3, detected by CCK-8 assay. (B) Proliferative ability of Eca109 and TE1 cells was significantly decreased in shRNA-Id3 cells by clone formation assay. (C) Silence of Id3 inhibited ESCC cell migration *in vitro* as assessed by the wound healing assay (scale bar, 100μm). (D) Silence of Id3 inhibited ESCC cell migratory and invasive abilities *in vitro* as assessed by the transwell assay (scale bar, 100 μm) *P<0.05, **P<0.01.

To further elucidate the biological impact of Id3 on ESCC cells, Id3 expression was silenced using a lentiviral vector carrying the shRNA targeting Id3 (shId3#1, shId3#2, Fig. S2B). The results showed that Id3 knockdown markedly suppressed the proliferative (Fig. 4A, 4B), migratory and invasive abilities in ESCC cells (Fig. 4C, 4D).

### Id3 promoted EMT and the tumorgenicity *in vivo*

**Figure 5:**
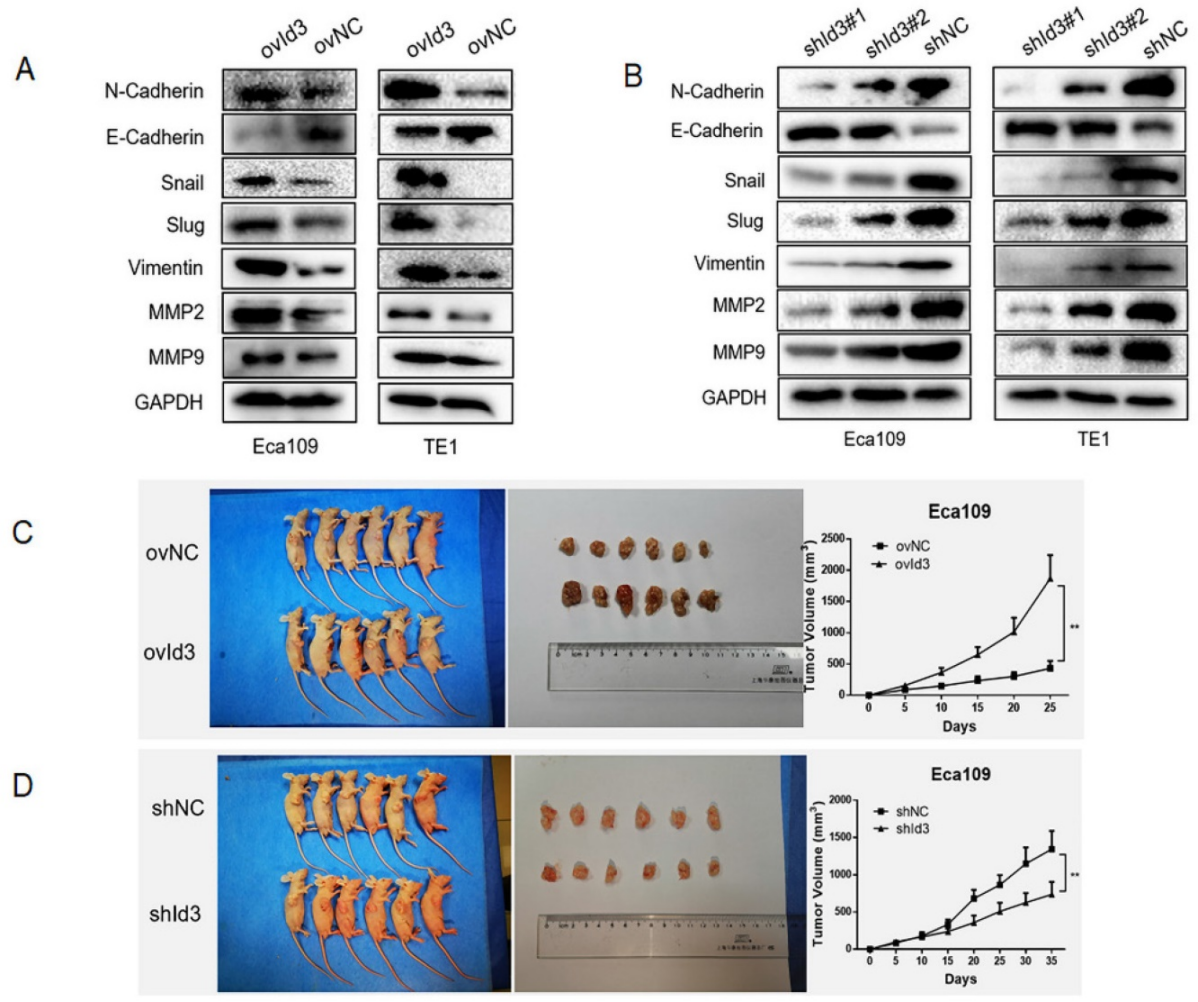
Id3 promoted EMT of ESCC cells and the tumorgenicity *in vivo*. (A) Overexpression of Id3 increased the expression of N-cadherin, Snail, Slug, Vimentin, MMP2, MMP9 while decreased the expression of E-cadherin in ESCC cells. (B) Silencing Id3 decreased the expression of N-cadherin, Snail, Slug, Vimentin, MMP2, MMP9 while increased the expression of E-cadherin in ESCC cells. (C, D) ovId3 cells promoted tumor growth (C) and shId3 cells inhibited tumor growth (D) *in vivo*. *P<0.05, **P<0.01.

Next, we evaluated the effects of Id3 on the EMT of ESCC cells. The results of WB revealed that after the overexpression of Id3, expression of epithelial marker (E-cadherin) was decreased while expression of mesenchymal markers, such as N-cadherin, Snail, Slug, Vimentin, MMP2, and MMP9 was increased (Fig. 5A). On the contrary, increased epithelial marker (E-cadherin) whereas decreased mesenchymal marker were observed in the shId3 group (Fig. 5B), indicating that Id3 overexpression could enhance EMT capacity of ESCC cells. To further validate the oncogenic role of Id3, we inoculated ovId3 or shId3 Eca109 cells into the nude mice. Results showed that tumors of ovId3 group were larger than those from control group, while shId3 group had smaller volume of tumors than control group (Fig. 5C, 5D).

### ERK/MAPK signaling pathway played an important role in Id3-mediated migration, invasion and EMT in ESCC cells

**Figure 6:**
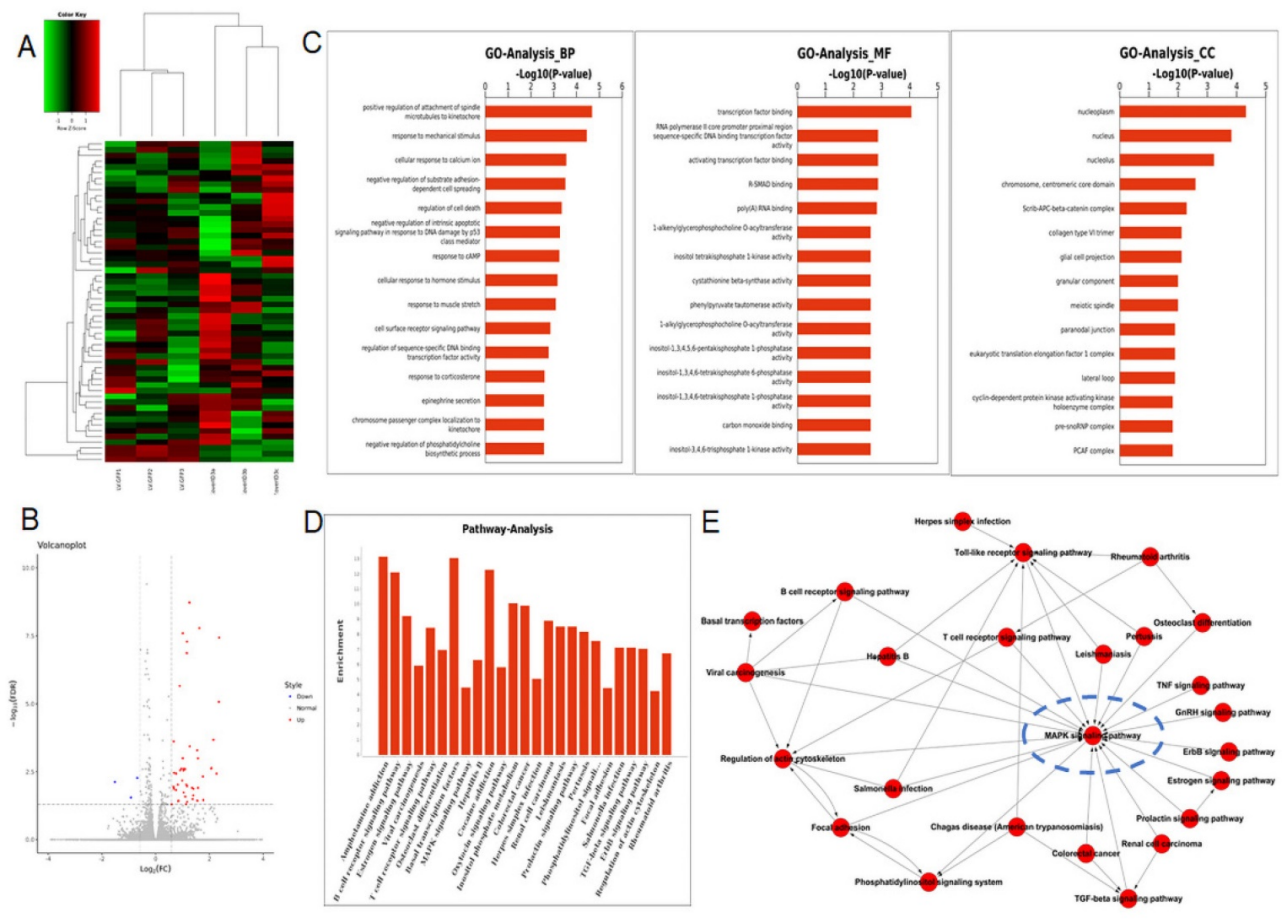
RNA-seq analysis data after Id3 was overexpressed in Eca109 cells. (A, B) Heatmap and volcano plot of differentially expressed transcripts. (C, D) Gene ontology analysis (C) and pathway analysis (D) based on all identified transcripts. (E) Pathway-act network of all pathways according to pathway database.

To figure out the underlying mechanism by which Id3-mediated oncogenic roles in ESCC, RNA-Seq analysis was applied in ovId3 Eca109 cells and scramble control Eca109 cells. Differentially expressed genes were analyzed and displayed in Fig. 6A and Fig. 6B. Gene ontology (GO) analysis included biological processes, molecular functions, and cell components was revealed in Fig. 6C. Based on these results, Id3 may regulated the cell cycle, cell death, differentiation and so on. In addition, potential pathways and pathway-act networks related to Id3 was also displayed (Fig. 6D, E).

Several studies have revealed that Id-protein induced cell proliferation was associated with the activation of ERK/MAPK signaling pathway 20,21. Among the all pathways of the RNA-Seq analysis, MAPK signaling pathway was significantly dysregulated in Id3-overexpressing Eca109 cells (Fig. 6D). Pathway-act network also indicated that the MAPK signaling pathway was located in the core area of the pathway map (Fig. 6E), which suggest that Id3 may have impacts on this pathway.

**Figure 7:**
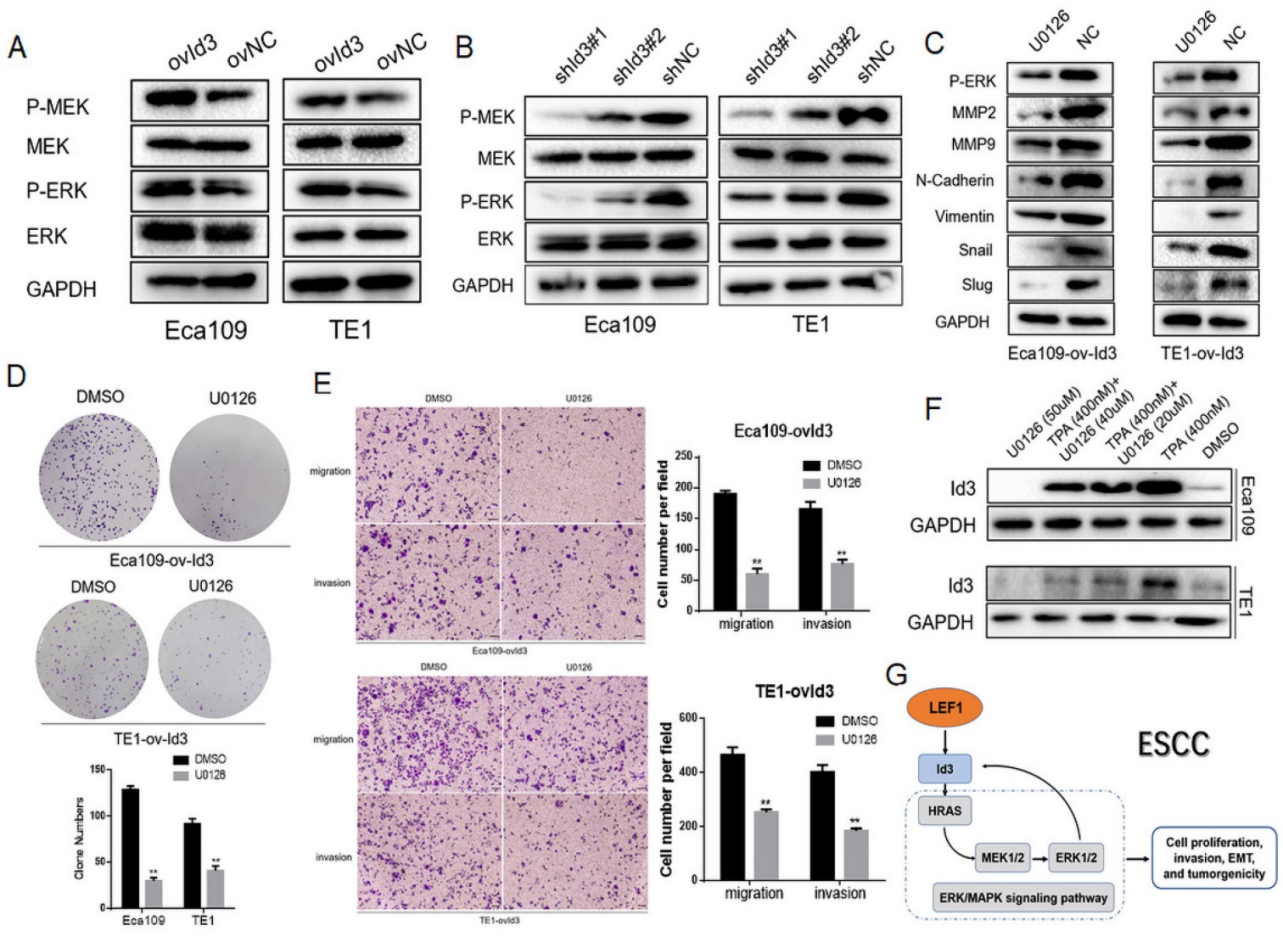
An interaction between Id3 and ERK/MAPK signaling pathway. (A, B) Western blots comparing the expression levels of P-ERK1/2, ERK, P-MEK1/2, and MEK1/2 in Id3-overexpressing and Id3-silenced cells with their control cells (NC). (C) U0126 attenuated the expression of EMT markers in ovId3 ESCC cells. (D) U0126 attenuated the proliferative ability of ovId3 ESCC cells. (E) U0126 attenuated the migratory and invasive abilities of ovId3 cells. (F) ERK/MAPK signaling pathway regulated the expression of Id3 in ESCC cells. (G) Schematic diagram of a positive feedback loop between Id3 and ERK/MAPK signaling pathway. *P<0.05, **P<0.01

The MAPK family consists of ERK, p38 and JNK pathway. Thus, we detected the core proteins expression of these pathways, including p-MEK1/2, MEK1/2, p-ERK1/2, ERK1/2, p-p38, p38, p-JNK, and JNK. As shown in Fig. 7A, 7B, Id3 overexpression increased the expressions of p-MEK1/2 and p-ERK1/2 while the knockdown of Id3 decreased the expressions of p-MEK1/2 and p-ERK1/2 in both Eca109 and TE1 cells, indicating Id3 may regulate the ERK/MAPK pathway. However, no significant protein changes were observed of p38 or JNK pathway (data not shown).

To further verify the biological functions of Id3 was through activating the ERK/MAPK pathway, we utilized the U0126, an antagonist of ERK/MAPK pathway, to measure whether it attenuated the effect of Id3. WB analysis validated that U0126 could effectively decrease expression levels of p-ERK1/2 (Fig. 7C). Colony forming ability of ovId3 ESCC cells treated with U0126 was reduced (Fig. 7D). Moreover, U0126 significantly compromised the migratory and invasive abilities of ovId3 cells (Fig. 7E, Fig S2C). Consistently, examining EMT markers demonstrated that U0126 treatment repressed the expression of MMP2, MMP9, N-cadherin, Vimentin, Snail, and Slug in Id3-overexpressing ESCC cells (Fig. 7C).

Previous study revealed that activating ERK/MAPK pathway in thymocytes could induce the expression of Id3 22. Whether this effect exists in ESCC cells is unclear. Thus, we incubated ESCC cells with U0126 and TPA (an activator of ERK/MAPK pathway). Interestingly, Id3 expression was increased after TPA treatment, whereas it was decreased after U0126 treatment, indicating that ERK/MAPK pathway could induce the expression of Id3 in ESCC cells (Fig. 7F).

Altogether, we discovered ERK/MAPK signaling pathway was essential for Id3-mediated biological functions in ESCC cells. Besides, ERK/MAPK pathway also regulate Id3 expression in turn. A local positive feedback loop may exist between Id3 and ERK/MAPK pathway (Fig. 7G).

### Id3 regulated MAPK signaling pathway via activating the expression of HRAS

**Figure 8:**
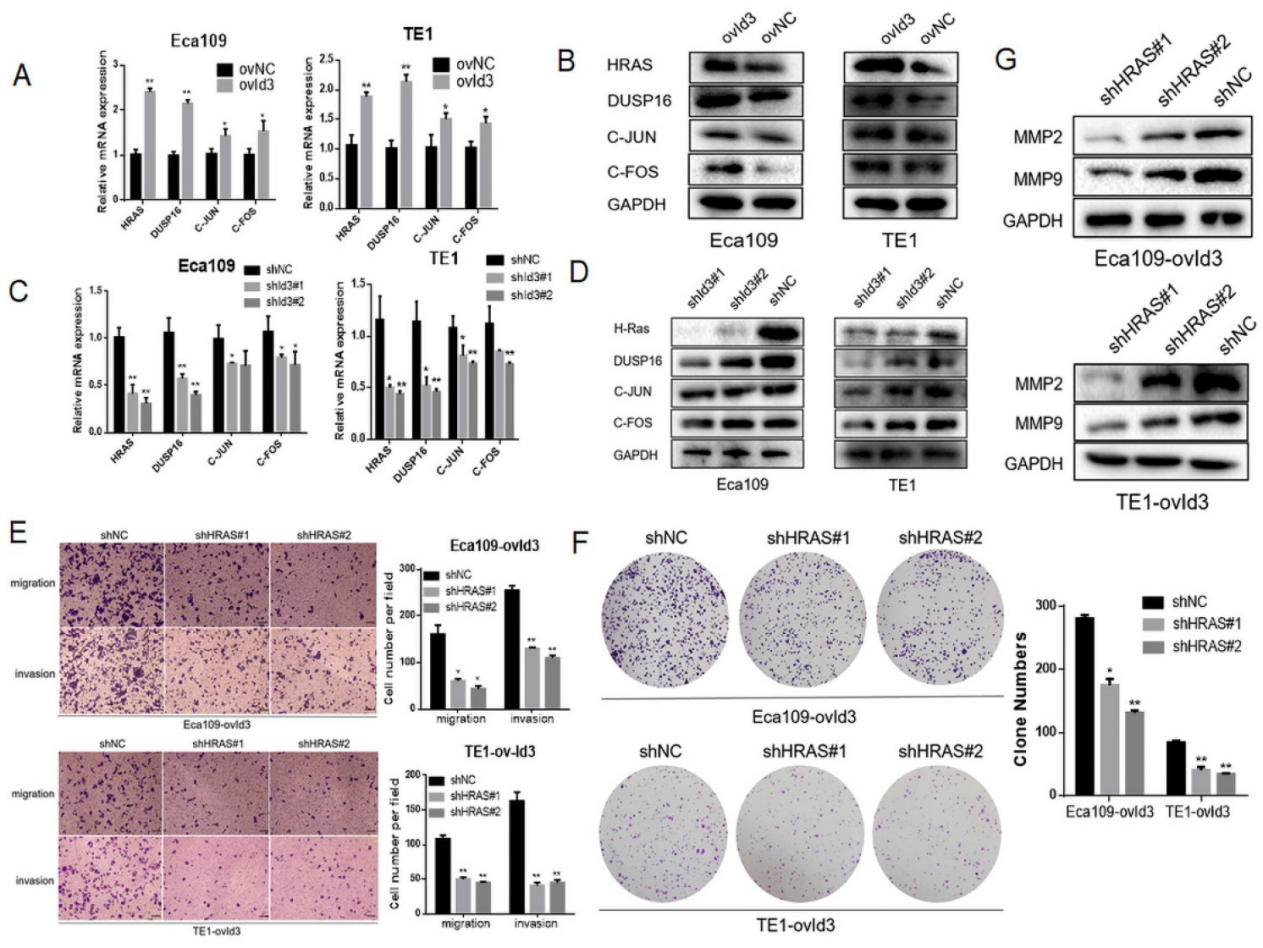
Id3 regulated MAPK signaling pathway via activating the expression of HRAS. (A, B) The expression levels of four differentially expressed genes were validated by qRT-PCR and western blotting analysis in ovId3 ESCC cells verse NC cells. (C, D) The expression levels of four differentially expressed genes were validated by qRT-PCR and western blotting analysis in shId3 ESCC cells verse NC cells. (E, F) Knockdown of HRAS diminished the effects of migration, invasion (E) and proliferation (F) in ovId3 ESCC cells. (G) Knockdown of HRAS reversed the EMT markers in ovId3 ESCC cells. *P<0.05, **P<0.01.

Based on the RNA-Seq data, four differentially expressed genes were enriched in the MAPK pathway, including HRAS, DUSP16, FOS and JUN. qRT-PCR and western blotting analysis measured the expression of these genes, which further validated the reliability of our RNA-Seq analysis (Fig. 8A-D). Among the four genes, HRAS and DUSP16 showed the most obviously changes after Id3 was altered. On the ground that RAS is the upstream gene and a key regulator of ERK/MAPK pathway, it is justifiable to conjecture that Id3 induces MEK/ERK/MAPK cascades via activating the expression of HRAS in ESCC. To validate this speculation, we applied a rescue strategy by downregulating HRAS in ovId3 ESCC cells. As expected, transwell assay and colony forming assay demonstrated that knockdown of HRAS could diminish the impacts of Id3 on migration, invasion and proliferation (Fig. 8E, 8F). Correspondingly, EMT markers were reversed due to the silencing HRAS in ovId3 ESCC cells (Fig. 8G). Taken together, these results verified that Id3 regulated ERK/MAPK signaling pathway via activating the expression of HRAS.

## Discussion

According to our previous and present studies, LEF1 could directly bind to the promoter of both Id1 and Id3. By Pearson's correlation analysis, LEF1 preferentially regulated the expression of Id3 than Id1 in terms of protein level. A luciferase reporter assay and ChIP assay identified the LEF1-binding site was located at -1100 to -1114 of the Id3 promoter. This result drove us to select Id3 for further study.

In humans, Id1 gene is located on chromosomes 20q11 and Id3 gene is located on chromosomes 1p36.1 9. Increased Id1 and Id3 expression is observed in multiple cancers and predicts a poor prognosis, including breast cancer, prostate cancer, colorectal cancer, et al. Although Id1 and Id3 are reported compensatory in the mouse model 23, several studies believe that Id1 and Id3 could have distinct pathways 24.

The research about Id1 and Id3 in ESCC is limited. Li et al found that Id1 promoted the tumorigenicity and metastasis of ESCC through activation of PI3K/AKT pathway 25,26. Id1 is overexpressed in ESCC tissues and may be used for prognostication for ESCC patients 27-29. Compared with Id1 and its significance in ESCC, Id3 is much less investigated. In this study, we revealed that Id3 was highly expressed in ESCC specimens and negatively associated with OS of ESCC patients. Moreover, Id3 could enhance the proliferation, migration, invasion and EMT capacity *in vitro* and promote the growth of tumors *in vivo*. All above findings are original and suggest that Id3 may be a novel target for ESCC treatment.

The downstream regulatory mechanism of Id3 in cancers is various. Id3 could promote the stemness of intrahepatic cholangiocarcinoma by increasing the transcriptional activity of Wnt/β-catenin signaling 30. Chen et al argued that Id3 could reverse cisplatin resistance in lung adenocarcinoma cells via regulating the PI3K/Akt pathway 31. In addition, Chen et al found that Id3 induced apoptosis in squamous carcinoma cells by an Elk-1-caspase-8-dependent pathway 32. Based on the RNA-Seq analysis, the MAPK signaling pathway was showed significantly dysregulated after Id3 was changed in Eca109 cells. It is widely recognized that RAS/MEK/ERK signaling pathway is one of the most commonly deregulated pathways, which plays critical role in regulating cell growth and tumorigenesis in various cancers, including ESCC 33,34. Therefore, it is conceivable that Id3 may regulate the MAPK pathway in ESCC. To test the effect of Id3 on this pathway, WB was used to study the expression of the key factors of the signaling pathway. As expected, the proteins of ERK/MAPK signaling pathway were increased in ovId3 cells while decreased in shId3 cells. Furthermore, the blockage of ERK/MAPK pathway by U0126 attenuated the biological effect of Id3 on ESCC cells, verifying the presumption that the effect of Id3 on migration, invasion and EMT was mediated by ERK/MAPK signaling pathway in ESCC cells.

Previous studies have confirmed that Id1 is not only an upstream gene but also a downstream target of the MAPK signaling pathway, implying a positive feedback loop exists between Id1 and MAPK signaling pathway 20,21. In this study, we firstly demonstrated that Id3 could regulate the ERK/MAPK pathway in ESCC cells. More interestingly, Id3 gene was proved to be a downstream target of the ERK/MAPK pathway in ESCC cells, which were indeed consistent with previous report 22. This means that a positive regulatory loop between Id3 and ERK/MAPK pathway may exist in ESCC.

Knockdown of HRAS or inhibition of MEK could reduce cell growth in mutant HRAS ESCC cell lines 35. Several studies have revealed that ectopic expression of HRAS led to enhanced ability of cell migration, invasion, EMT and metastasis in breast cancer and colorectal cancer, suggesting that HRAS could be an important factor responsible for the progression of cancer 36,37. In our study, we observed that Id3 could regulate the expression of HRAS in ESCC cells. Given that Id3 lacks the DNA-binding motif and performs its biological function via protein-protein interactions with other bHLH proteins 38, it is plausible that Id3 regulates the HRAS expression through an indirect manner. By a rescue experiment, the depletion of HRAS abolished the Id3 mediated biological functions in ESCC cells, indicating that Id3 promoted ERK/MAPK pathway in ESCC cells is in HRAS dependent manner.

In conclusion, our study demonstrated that targeting LEF1/Id3/HRAS axis may suppress ESCC cell growth, migration, invasion and EMT via blocking ERK/MAPK signaling pathway. Blocking this axis may have promising therapeutic potential to delay the progress of ESCC.

## Supplementary Material

Supplementary figures and tables.

## Figures and Tables

**Table 1:** Correlation between LEF1 and Id3 expression in ESCC tissues.

		LEF1 expression		
		Low expression	High expression	P value
Id3 expression	Low expression	17 (16.30%)	16 (18.48%)	**<0.01**
	High expression	7 (9.78%)	52 (55.43%)	

**Table 2:** Expression of Id3 in ESCC tumor tissues and corresponding adjacent normal tissues.

		Id3 expression		
	Cases	Low expression	High expression	P value
Tumor tissues	92	33(35.87%)	59(64.13%)	**<0.01**
Normal tissues	92	67(72.83%)	25(27.17%)	

**Table 3:** Correlations between Id3 expression and clinicopathologic features in ESCC.

	Id3 expression, n (%)		
Characteristic	Low expression	High expression	P value
Age			0.500
<65	23 (38.33%)	37 (61.67%)	
≧65	10 (31.25%)	22 (68.75%)	
Sex			0.572
Male	24 (34.29%)	46 (65.71%)	
Female	9 (40.91%)	13 (59.09%)	
Tumor location			0.907
Upper	2 (28.57%)	5 (71.43%)	
Middle	20 (37.04%)	34 (62.96%)	
Lower	11 (35.48%)	20 (64.52%)	
Histologic differentiation			**0.011**
Grade 1	15 (55.56%)	12 (44.44%)	
Grade 2-3	18 (27.69%)	47 (72.31%)	
Pathological T stage			**<0.01**
T1-2	19 (59.38%)	13 (40.62%)	
T3-4	14 (23.33%)	46 (76.67%)	
Pathological N stage			0.074
N0-1	28 (41.18%)	40 (58.82%)	
N2-3	5 (20.83%)	19 (79.17%)	
Pathologic staging			**<0.01**
I-II	22 (55.00%)	18 (45.00%)	
III-IV	11 (21.15%)	41 (78.85%)	

**Table 4:** Univariate and multivariate survival analysis for patients with ESCC.

Characteristic	HR	95% CI	P value
Univariate analysis			
Age (<65 vs≧65)	1.457	0.813-2.610	0.206
Sex (Male vs Female)	0.674	0.355-1.278	0.226
Location (Upper/Middle vs Lower)	1.032	0.565-1.888	0.918
Differentiation (Grade 1 vs Grade 2/3)	2.191	1.059-4.536	**0.035**
pT category (T1-2 vs T3-4)	1.549	0.817-2.936	0.180
pN category (N0-1 vs N2-3)	1.997	1.089-3.664	**0.025**
Id3 expression (Low vs High)	3.243	1.566-6.718	**0.002**
Multivariate analysis			
Differentiation (Grade 1 vs Grade 2/3)	1.645	0.779-3.473	0.192
pN category (N0-1 vs N2-3)	1.513	0.813-2.814	0.191
Id3 expression (Low vs High)	2.776	1.321-5.830	**0.007**

95% CI = 95% confidence interval; HR = Hazard Risk.

## Abbreviations

EC: Esophageal cancer
ESCC: esophageal squamous cell carcinoma
LEF1: Lymphoid enhancer-binding factor 1
OS: overall survival
qRT-PCR: Quantitative Real-Time Polymerase Chain Reaction
EMT: epithelial-mesenchymal transition

## References

[1] Siegel RL, Miller KD, Jemal A (2018). Cancer statistics, 2018. CA Cancer J Clin.

[2] Rustgi AK, El-Serag HB (2014). Esophageal carcinoma. N Engl J Med.

[3] Santiago L, Daniels G, Wang D (2017). Wnt signaling pathway protein LEF1 in cancer, as a biomarker for prognosis and a target for treatment. Am J Cancer Res.

[4] Delaunay S, Rapino F, Tharun L (2016). Elp3 links tRNA modification to IRES-dependent translation of LEF1 to sustain metastasis in breast cancer. J Exp Med.

[5] Huang LX, Hu CY, Jing L (2017). microRNA-219-5p inhibits epithelial-mesenchymal transition and metastasis of colorectal cancer by targeting lymphoid enhancer-binding factor 1. Cancer Sci.

[6] Li Y, Wang L, Zhang M (2009). LEF1 in Androgen-Independent Prostate Cancer: Regulation of Androgen Receptor Expression, Prostate Cancer Growth, and Invasion. Cancer Res.

[7] Zhao Y, Li C, Huang L (2018). Prognostic value of association of OCT4 with LEF1 expression in esophageal squamous cell carcinoma and their impact on epithelial-mesenchymal transition, invasion, and migration. Cancer Med.

[8] Wang X, Zhao Y, Lu Q (2020). MiR-34a-5p Inhibits Proliferation, Migration, Invasion and Epithelial-mesenchymal Transition in Esophageal Squamous Cell Carcinoma by Targeting LEF1 and Inactivation of the Hippo-YAP1/TAZ Signaling Pathway. J Cancer.

[9] Zhao Y, Zhu J, Shi B (2019). The transcription factor LEF1 promotes tumorigenicity and activates the TGF-β signaling pathway in esophageal squamous cell carcinoma. J Exp Clin Cancer Res.

[10] Roschger C, Cabrele C (2017). The Id-protein family in developmental and cancer-associated pathways. Cell Commun Signal.

[11] Ahlqvist K, Saamarthy K, Syed Khaja AS, Bjartell A, Massoumi R (2013). Expression of Id proteins is regulated by the Bcl-3 proto-oncogene in prostate cancer. Oncogene.

[12] Lasorella A, Benezra R, Iavarone A (2014). The ID proteins: master regulators of cancer stem cells and tumour aggressiveness. Nat Rev Cancer.

[13] Sharma BK, Kolhe R, Black SM, Keller JR, Mivechi NF, Satyanarayana A (2016). Inhibitor of differentiation 1 transcription factor promotes metabolic reprogramming in hepatocellular carcinoma cells. FASEB J.

[14] Gumireddy K, Li A, Gimotty PA (2009). KLF17 is a negative regulator of epithelialmesenchymal transition and metastasis in breast cancer. Nat Cell Biol.

[15] Antonângelo L, Tuma T, Fabro A (2016). Id-1, Id-2, and Id-3 co-expression correlates with prognosis in stage I and II lung adenocarcinoma patients treated with surgery and adjuvant chemotherapy. Exp Biol Med (Maywood).

[16] Fernandez-Medarde A, Santos E (2011). Ras in cancer and developmental diseases. Genes Cancer.

[17] Umstead M, Xiong J, Qi Q, Du Y, Fu H (2017). Aurora kinase A interacts with H-Ras and potentiates Ras-MAPK signaling. Oncotarget.

[18] Bahrami A, Hassanian SM, ShahidSales S (2018). Targeting RAS signaling pathway as a potential therapeutic target in the treatment of colorectal cancer. J Cell Physiol.

[19] Cristea S, Sage J (2016). Is the Canonical RAF/MEK/ERK Signaling Pathway a Therapeutic Target in SCLC?. J Thorac Oncol.

[20] Ling MT, Wang X, Ouyang XS (2002). Activation of MAPK signaling pathway is essential for Id-1 induced serum independent prostate cancer cell growth. Oncogene.

[21] Tournay O, Benezra R (1996). Transcription of the dominant-negative helix-loophelix protein Id1 is regulated by a protein complex containing the immediate-early response gene Egr-1. Mol Cell Biol.

[22] Bain G, Cravatt CB, Loomans C, Alberola-Ila J, Hedrick SM, Murre C (2001). Regulation of the helix-loop-helix proteins, E2A and Id3, by the Ras-ERK MAPK cascade. Nat Immunol.

[23] Lyden D, Young AZ, Zagzag D, Yan W, Gerald W, O'Reilly R (1999). Id1 and Id3 are required for neurogenesis, angiogenesis and vascularization of tumour xenografts. Nature.

[24] Perry SS, Zhao Y, Nie L, Cochrane SW, Huang Z, Sun XH (2007). Id1, but not Id3, directs long-term repopulating hematopoietic stem-cell maintenance. Blood.

[25] Li B, Cheung P, Wang X (2007). Id-1 activation of PI3K/Akt/NFκB signaling pathway and its significance in promoting survival of esophageal cancer cells. Carcinogenesis.

[26] Li B, Tsao SW, Li YY (2010). Id-1 promotes tumorigenicity and metastasis of human esophageal cancer cells through activation of PI3K/AKT signaling pathway. Int J Cancer.

[27] Luo KJ, Wen J, Xie X (2012). Prognostic relevance of Id-1 expression in patients with resectable esophageal squamous cell carcinoma. Ann Thorac Surg.

[28] Liu J, Hu Y, Hu W, Xie X, Ela Bella A, Fu J (2010). Expression and prognostic relevance of Id1 in stage III esophageal squamous cell carcinoma. Cancer Biomark.

[29] Yuen HF, Chan YP, Chan KK (2007). Id-1 and Id-2 are markers for metastasis and prognosis in oesophageal squamous cell carcinoma. Br J Cancer.

[30] Huang L, Cai J, Guo H (2019). ID3 Promotes Stem Cell Features and Predicts Chemotherapeutic Response of Intrahepatic Cholangiocarcinoma. Hepatology.

[31] Chen FF, Lv X, Zhao QF (2018). Inhibitor of DNA binding 3 reverses cisplatin resistance in human lung adenocarcinoma cells by regulating the PI3K/Akt pathway. Oncol Lett.

[32] Chen YS, Aubee J, DiVito KA (2015). Id3 induces an Elk-1-caspase-8-dependent apoptotic pathway in squamous carcinoma cells. Cancer Med.

[33] Hu X, Zhai Y, Kong P (2017). FAT1 prevents epithelial mesenchymal transition (EMT) via MAPK/ERK signaling pathway in esophageal squamous cell cancer. Cancer Lett.

[34] Chen L, Bi S, Hou J, Zhao Z, Wang C, Xie S (2019). Targeting p21-activated kinase 1 inhibits growth and metastasis via Raf1/MEK1/ERK signaling in esophageal squamous cell carcinoma cells. Cell Commun Signal.

[35] Kiessling MK, Curioni-Fontecedro A, Samaras P (2015). Mutant HRAS as novel target for MEK and mTOR inhibitors. Oncotarget.

[36] Vuoriluoto K, Haugen H, Kiviluoto S (2011). Vimentin regulates EMT induction by Slug and oncogenic H-Ras and migration by governing Axl expression in breast cancer. Oncogene.

[37] Liu Z, Liu J, Dong X (2019). Tn antigen promotes human colorectal cancer metastasis via H-Ras mediated epithelial-mesenchymal transition activation. J Cell Mol Med.

[38] Langlands K, Yin X, Anand G, Prochownik EV (1997). Differential interactions of Id proteins with basic-helix-loop-helix transcription factors. J Biol Chem.

