# Supplementary material for: LEF1/Id3/HRAS axis promotes the tumorigenesis and progression of esophageal squamous cell carcinoma
Source: Int J Biol Sci. 2020 Jun 29;16(13):2392–404. doi: 10.7150/ijbs.47035 (PMC7378645; doi:10.7150/ijbs.47035)
Supplement: Supplementary file 1 — Supplementary figures and tables. [file ijbsv16p2392s1.pdf]

Supporting Information

Supporting Fig. S1 Correlation of Id3 expression and histologic differentiation and lymph node metastasis in the UALCAN database. (A) Id3 expression level was associated with histologic differentiation in esophageal cancer. (B) Patients with the lymph node metastasis tended to have higher Id3 expression.

Supporting Fig. S2 The mRNA and protein level of Id3 was detected after transfected with overexpressed Id3 (A) or shRNA-Id3 (B) in both Eca109 and TE1 cells. (C) The wound healing assay showed that U0126 inhibited the migratory abilities in ovId3 ESCC cells. *$P<0.05$, **$P<0.01$

Fig. S1

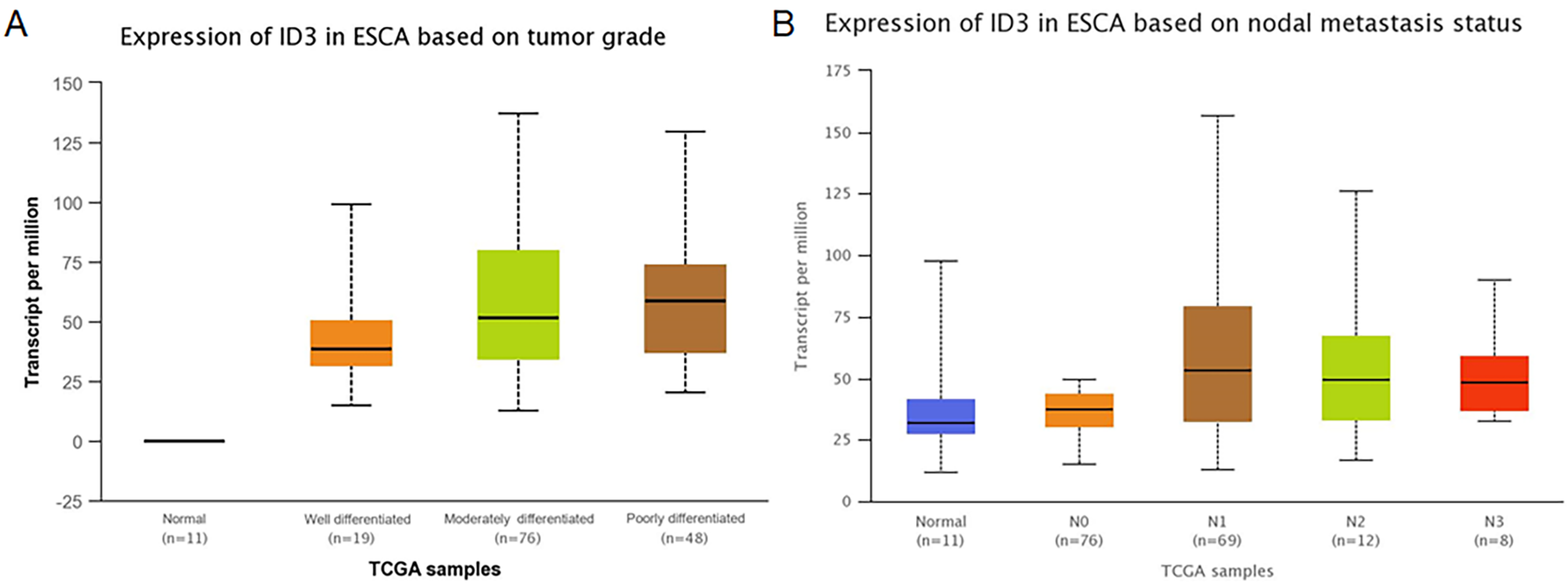

A
Expression of ID3 in ESCA based on tumor grade
150
125
100
75
50
25
0
-25
Transcript per million
Normal
(n=11)
Well differentiated
(n=19)
Moderately differentiated
(n=76)
Poorly differentiated
(n=48)
TCGA samples
B
Expression of ID3 in ESCA based on nodal metastasis status
175
150
125
100
75
50
25
0
Transcript per million
Normal
(n=11)
N0
(n=76)
N1
(n=69)
N2
(n=12)
N3
(n=8)
TCGA samples

Fig. S2

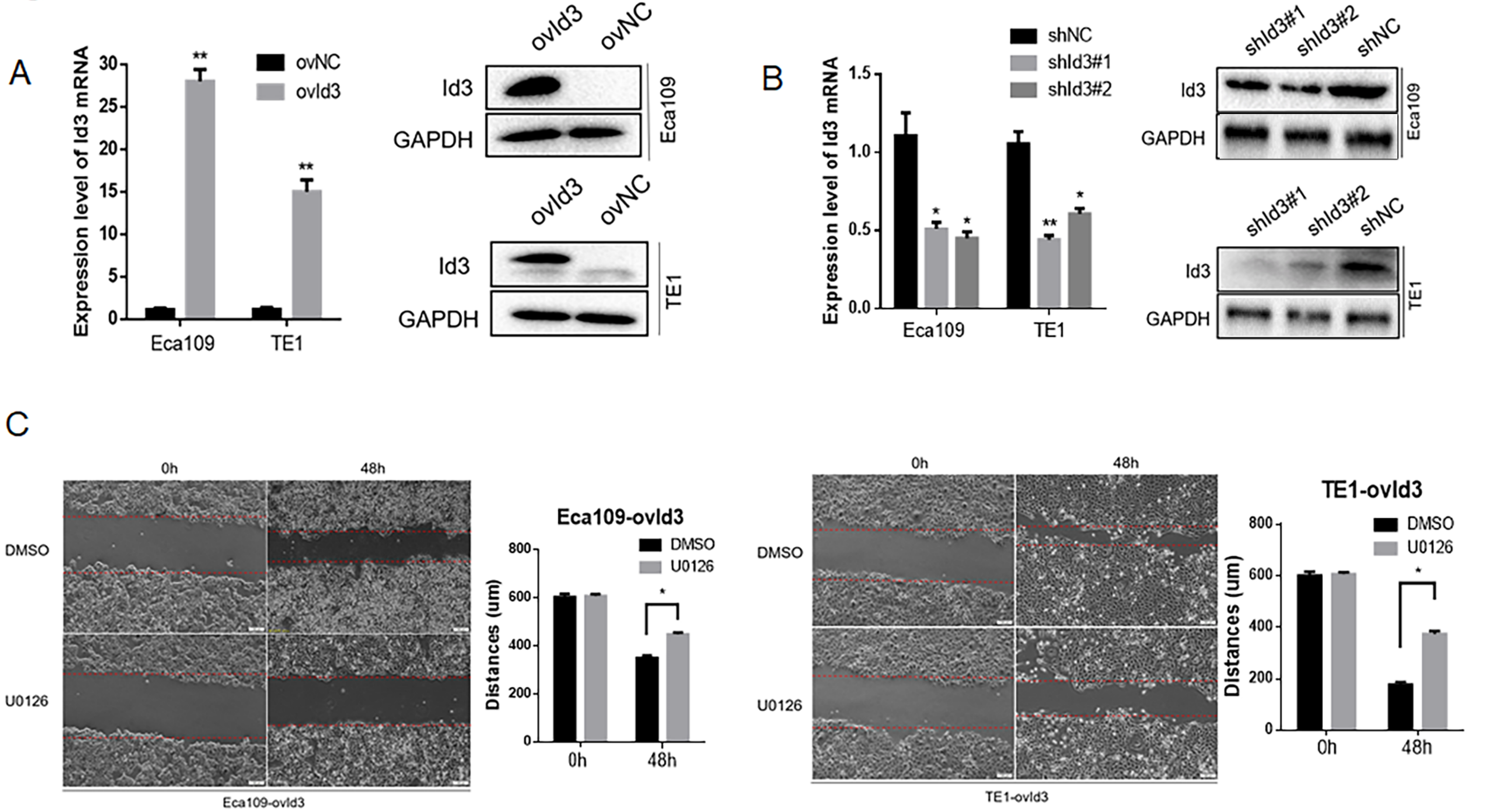
A
Expression level of Id3 mRNA
30
25
20
15
10
5
0
**
**
ovNC
ovId3
Eca109
TE1
ovId3
ovNC
Id3
GAPDH
Eca109
TE1
B
Expression level of Id3 mRNA
1.5
1.0
0.5
0.0
shNC
shId3#1
shId3#2
Eca109
TE1
shId3#1
shId3#2
shNC
Id3
GAPDH
C
0h
48h
DMSO
U0126
Eca109-ovId3
Distances (um)
800
600
400
200
0
*
TE1-ovId3
